# Maturity-dependent changes in the nutritional, functional, mineral, and structural properties of dragon fruit peel (*Selenicereus* spp.)

**DOI:** 10.3389/fpls.2026.1893418

**Published:** 2026-08-17

**Authors:** Mangalambika Balaji, Utpal Das, Rahul Vashishth

**Affiliations:** 1 Department of Horticulture and Food Science, Vellore Institute of Technology (VIT) School of Agriculture Innovations and Advanced Learning, Vellore Institute of Technology, Vellore, Tamil Nadu, India; 2 Department of Biosciences, School of Biosciences and Technology, Vellore Institute of Technology, Vellore, Tamil Nadu, India

**Keywords:** antioxidant activity, dragon fruit peel, functional properties, mineral composition, nutritional composition, *Selenicereus* spp., structural characterization

## Abstract

**Introduction:**

Dragon fruit peel (*Selenicereus spp*.), a major by-product of fruit processing, is a source of dietary fibre, minerals, and bioactive compounds; However combined effect of maturity stage and variety on nutritional, functional, antioxidant, mineral, anti-nutritional and structural properties of dragon fruit peel is limited. Thus, this study aimed to analyze the physicochemical properties, antioxidant activities, anti-nutritional activities, functional properties, mineral and structural analysis of dragon fruit peel powder obtained from different maturity stages and varieties.

**Methods:**

Dragon fruit peels from four varieties (C, CM Pink, Taiwan Pink and Jumbo Red) at three maturity stages (25th,35th and 45th days after anthesis; DAA) were hot-air dried and analyzed for proximate composition, antioxidant activity [2,2-diphenyl-1-picrylhydrazyl (DPPH), 2,2′-azino-bis(3-ethylbenzothiazoline-6-sulfonic acid) (ABTS), and ferric reducing antioxidant power (FRAP)], anti-nutritional factors, mineral composition, and functional properties. Structural characteristics were evaluated using scanning electron microscopy (SEM), Fourier transform infrared spectroscopy (FTIR), and X-ray diffraction (XRD).

**Results:**

The functional properties, such as oil absorption capacity, water-holding capacity, and swelling capacity also improved with maturity, from 1.33–1.66 g/g, 1.12– 1.13 g/mL, and 3.43–4.19 g/g, respectively, to 2.66–3.97 g/g, 2.05–2.31 g/mL, and 6.20–6.32 g/g, respectively. Dietary fibre and mineral contents increased with maturity, whereas total phenolic content, flavonoid content, and antioxidant activities declined. Phytic acid decreased, while oxalate and tannin contents increased moderately. The results of structural analyses showed a higher porosity and maturational changes in the functional groups and crystallinity.

**Discussion:**

Overall, mature dragon fruit peel had better nutritional and technological functionality, making it suitable as a food ingredient with additional fibre and mineral contents. In addition, bioavailability, digestibility, safety and formulation evaluation studies are needed to enable use in functional food systems.

## 1 Introduction

Dragon fruit (*Selenicereus* spp.) belongs to the family Cactaceae. It has emerged as an important commercial fruit crop in tropical and subtropical regions, including Asia, Central America, and other tropical areas (Jamilah et al., 2011; Wichienchot et al., 2010). Although native to Central and South America, this climbing cactus has adapted successfully to diverse agroclimatic conditions, with substantial production reported in Vietnam, Thailand, Malaysia, and India (Duncan et al., 2021; Mizrahi et al., 1996; Ortiz-Hernández and Carrillo-Salazar, 2012). Based on its morphology and taxonomy, dragon fruit can be classified under *Selenicereus*. These species have previously been included under the genus *Hylocereus*, and the earlier classification remains in use in a number of recent publications and in earlier publications. For this reason, both names continue to appear in the literature. However, *Selenicereus* is accepted as the genus (Korotkova et al., 2017). The fruit is distinguished by its bright pink or yellow peel with green scales and white or deep magenta flesh, dependent on the variety (Le Bellec and Vaillant, 2011). In addition to its attractive appearance, dragon fruit has gained considerable scientific and commercial attention owing to its abundance of bioactive compounds, including betalains, polyphenols, flavonoids, and ascorbic acid, which contribute to its antioxidant potential and health-promoting properties (Wu et al., 2006; Wybraniec and Mizrahi, 2002). Despite increasing consumption of dragon fruit pulp, the peel fraction, which accounts for approximately 30%–35% of the total fruit weight, remains largely underutilized and is commonly discarded as agricultural waste (Liaotrakoon et al., 2013; Tenore et al., 2012). This practice results in both economic and environmental post-harvest losses, particularly because dragon fruit peel contains substantial amounts of dietary fiber, minerals, phenolic compounds, and betalain pigments (Luo et al., 2014). Previous studies have reported that dragon fruit peel has higher antioxidant activity than the pulp (Handayani et al., 2018; Ramli et al., 2014; Strack et al., 2003), which is attributed to the presence of betalain pigments, betacyanins, and betaxanthins, which have free radical scavenging properties. The contribution of these pigments, however, may differ between varieties and maturity stages, which was not quantified in the present study.

In addition, the peel contains appreciable amounts of pectin and structural polysaccharides, making it a promising raw material for the development of functional food ingredients, natural colorants, and biodegradable packaging materials (Gunasena and Pushpakumara, 2007; Jalgaonkar et al., 2022; Hanan Taharuddin et al., 2023). In recent years, research work has focused on fruit processing by-products as valuable sustainable sources of dietary fiber, bioactive compounds, and functional ingredients for food applications. The high contents of phenolic compounds, pigments, dietary fiber, and minerals in dragon fruit peel have led to special attention for its possible nutritional enrichment and techno-functional improvement in food systems. In addition, progress in structural characterization and functional assessment has shown the need for knowledge on the maturity-dependent compositional changes to enable the best utilization of fruit-derived by-products. The results of this study highlight the importance of examining in detail the effects of variety and maturity on the quality attributes of dragon fruit peels (Kim et al., 2011; Sagar et al., 2018).

The utilization of agricultural by-products aligns with modern sustainability goals and circular economy principles, providing economic and environmental benefits while minimizing waste generation (Galanakis, 2012; Kim et al., 2011; Sagar et al., 2018; Lim et al., 2025). However,effective utilization of dragon fruit peel requires a comprehensive understanding of how variety and fruit maturity influence its nutritional composition, antioxidant activity, functional properties, and structural characteristics (Ariffin et al., 2009; Nerd et al., 1999; Tel-Zur, 2004). Biochemical composition, structural features, and postharvest quality are all influenced by fruit maturity, which in turn affects the suitability of fruit materials for nutritional and industrial uses (Quan et al., 2025). Therefore, understanding maturity-dependent changes is essential for optimizing the utilization of fruit processing by-products. Valorization of plant by-products as sustainable sources of bioactive compounds, dietary fiber, and functional ingredients has received increasing attention in recent research. The molecular structural characterization of plant polysaccharides and bioactive compounds has shown a strong correlation between molecular structure and biological or techno-functional properties (Hou et al., 2024; Gong et al., 2024). Moreover, the analytical techniques and methods developed have contributed to the understanding of the relationship between the compositional and structural features and the antioxidant properties, physicochemical functionality, and food applications of plant materials (Feng et al., 2025; Chang et al., 2025). These results indicate that the development of a comprehensive characterization of fruit processing by-products is essential to enable their utilization as valuable food ingredients. Current research has mainly concentrated on a few varieties, at a single maturity stage, or on a few compositional parameters of dragon fruit peel, which has been a valuable source of dietary fiber, minerals, pigments, and bioactive compounds. Hence, the interactive effects of variety and fruit maturity on the nutritional composition, antioxidant properties, nutritional functionality, presence of anti-nutrients, and structural properties of fruit peels are still not well understood. These data are needed in order to determine the best harvest periods and targeted use of the product for food and industrial applications. The availability of varieties with several distinct peel colors, fruit morphologies, and growth characteristics in India has made it a rapidly expanding dragon fruit producer (Kakade et al., 2025). In the present study, four varieties—C, CM Pink, Taiwan Pink, and Jumbo Red—were selected as they are commercially important varieties grown under Indian agro-climatic conditions and show significant variations in fruit size, peel proportion, fruit color, and fruit maturation behavior. These make them good models for the study of variety-dependent responses during fruit development. In addition, the rapid development of analytical technology has facilitated the understanding of how compositional and structural properties affect the antioxidant activity, physicochemical functionality, and possible applications of plant materials in the food industry (Feng et al., 2025; Quan et al., 2025; Chang et al., 2025). This study aimed to evaluate maturity-dependent changes in the quality attributes of dragon fruit peel across four commercially cultivated varieties. We also aimed to understand the potential applications of the peel by examining its composition, function, and structural characteristics.

## 2 Materials and methods

### 2.1 Sample collection and preparation

The experimental fruits were collected from the Dragon Fruits Farm located in Krishnagiri District, Tamil Nadu (12.55° N, 77.66° E; approximately 804 m above mean sea level), situated in the Northwestern Agro-Climatic Zone of Tamil Nadu, India. Four types of *Selenicereus* spp. [C, CM (CM Pink), T (Taiwan Pink), and J (Jumbo Red)], red skin and red flesh, were used in this study. The fruits were harvested at different maturity levels, i.e., 25th, 35th, and 45th day after anthesis (DAA). The selected varieties are commercial dragon fruit cultivars with varying fruit size, growth, and peel color. These varieties were chosen to evaluate maturity-dependent differences across genetic backgrounds and to determine whether there are differences within genotypic groups in terms of the nutritional, functional, antioxidant, mineral, anti-nutrient, and structural properties of dragon fruit peel. Preliminary observations indicated a progressive decline in fruit firmness and an increase in physiological loss in weight and decay incidence with advancing maturity. Fruits harvested beyond the 45th DAA showed accelerated softening, greater moisture loss, and increased decay susceptibility. Therefore, the 25th, 35th, and 45th DAA were selected to represent the immature, commercially mature, and advanced physiological maturity stages, respectively. These three maturity stages were selected based on published studies describing dragon fruit growth, ripening, and physiological development. These stages represent the immature, physiologically mature, and commercial type developmental stages, enabling evaluation of the compositional and structural changes during fruit maturation (Wanitchang et al., 2010). The developmental rates may vary among varieties, but all growing conditions were similar, and the selected stages are sufficient to represent maturity progression for the varieties of dragon fruit studied.

### 2.2 Physical properties of dragon fruit peel

The inedible index was determined as the percentage of non-pulp weight (peel, seeds, and other non-edible parts) from the total weight of the fruits. The fruit peel powder color parameter was measured using a COLOURFLEX EZ 45/0 spectrophotometer (Hunter Associates Laboratory Inc., Reston, VA, USA). Each measurement method was preceded by instrument calibration using standard white and black tiles. The color coding ranged along L* (lightness), a* (red–green axis), and b* (yellow–blue axis) as standardized processes for coordinating color (Cendrowski et al., 2023). The assessment was conducted at three positions on each fruit peel sample to accurately measure color. The pH of the fruit peel powder homogenate was measured directly using a calibrated digital pH meter for extraction (model 361; Systronics, Ahmedabad, India) at room temperature (25 ± 2°C) (Cendrowski et al., 2023).

### 2.3 Nutritional composition

Proximate composition (moisture, ash, protein, fat, and fiber) was determined following the AOAC (2010); AOAC (2019) standard methods. Carbohydrate content was calculated with the difference method using the formula: 100 − (moisture + ash + protein + fat + fiber). The total energy value was calculated using the formula: (protein × 4) + (fat × 9) + (carbohydrate × 4) kcal/100 g. The mineral composition was determined by ICP-MS (NexION 1000, PerkinElmer, Singapore, Singapore) at the SMEC-DST-PURSE Centre, Vellore Institute of Technology, following microwave-assisted digestion (Ahmad et al., 2021). The results are expressed as milligrams per 100 g dry weight (DW).

### 2.4 Functional properties of peel powder

The water holding capacity (WHC), oil absorption capacity (OAC), and swelling capacity (SC) of the peel powder were estimated using the method described by (Muhammad et al., 2020).

For the WHC, 100 mg of the peel powder sample was weighed and added into a 50-ml centrifuge tube containing 25 ml of distilled water. The mixture was incubated overnight at 4°C, centrifuged for 20 min at 13°C for 5,000 rpm, and left at room temperature for 2 h. The dried sample was weighed to obtain the dry residue weight. The WHC calculation is as follows: WHC (g/ml) = (Weight of hydrated sample − Initial dry weight of peel powder)/(Initial dry weight of peel powder).

A total of 100 mg of sample was added to a centrifuge tube holding 10 ml of vegetable oil to determine the OAC. The content was stirred and centrifuged at 3,500 rpm for 30 min. The supernatant was weighed and the OAC expressed as follows: OAC (g/g) = (Weight of absorbed oil − Initial dry weight of peel powder)/(Initial dry weight of peel powder).

For SC, the dragon fruit peel powder sample (100 mg) was weighed in a cone-shaped plastic tube. Distilled water (5 ml) was added to the tube and the mixture left overnight. The SC of the extracted sample was determined using the following equation: SC (g/g) = (Volume of swollen material (g) − Weight of dry powder (g))/(Weight of peel powder (g)).

### 2.5 Anti-nutritional activity

#### 2.5.1 Oxalate

The titrimetric method (Ee and Yates, 2013) was used to determine the oxalate content. Of the sample, 2 g was digested with 10 ml of 6 M hydrochloric acid, filtered, then titrated against standardized KMnO4 at 80°C until a slight pink color lasted for 30 s. The oxalate concentration was determined as the volume of KMnO_4_ used after subtracting the blank value. All dilution factors were considered, and the findings are reported as milligrams per 100 g sample. The results are expressed as milligrams per 100 g DW. To determine the content of oxalate, the calculation was done as follows: Oxalate (mg/100 g) = (*T* × *M*kMnO4 × 2.5 × *M*r × DF × 100)/(*W*), where *T* is the titer of KMnO_4_ (in milliliters), *M*_KMnO_4 is the molarity of KMnO_4_ (in moles per liter), *M*_r_ is the molar mass of oxalic acid (in grams per mole), DF is the dilution factor, and *W* is the weight of the sample (in grams).

#### 2.5.2 Phytate

Phytate phosphorus was determined according to the method of Griffiths (1982). HCl (2.4%) was used to extract 1 g of sample for 3 h. The resulting extract was reacted with the FeCl_3_ reagent, heated for 45 min, cooled, and then centrifuged. The precipitate was dissolved in 3.2 ml of 1.5 M NaOH, and absorbance was measured at 470 nm. A standard curve was used to measure the phytate content, accounting for the extract volume and dilution factors. The results are expressed as milligrams per 100 g DW. The phytate content of the sample was determined as follows. Phytate (mg/100 g) = (*C* × *V*ext × DF × 100)/(*W*), where *C* is the concentration from the standard curve (in milligrams per milliliter), *V*_ext_ is the volume of extract (in milliliter), DF is the dilution factor, and *W* is the weight of the sample (in grams).

#### 2.5.3 Alkaloid

Harborne (1973) procedure, with some modifications, was followed to estimate the amount of alkaloid. A 10% acetic acid in ethanol (1:10, *w*/*v*) solution was used to extract 2 g of the sample, which was left to stand for 4 h. The extract was reduced to a quarter of the total volume and the alkaloids precipitated. Concentrated ammonium hydroxide was added. The precipitate was filtered and dried at 60°C until it attained a constant weight. The results are expressed as milligrams per 100 g DW. The alkaloid content is expressed as milligrams per 100 g of the sample using the formula: Alkaloids (mg/100 g) = (*W*alk/*W*sample) × 100, where *W*alk is the weight of the dried alkaloid precipitate (in grams) and *W*sample is the initial weight of the sample (in grams).

#### 2.5.4 Total tannin content

Total tannin content was determined using the indigo carmine titration method as described in Hajimahmoodi et al. (2013) and Kumari (2015). An extract prepared from 5 g of dried dragon fruit peel powder was titrated against 0.1 N potassium permanganate (KMnO_4_) using indigo carmine as the indicator, with a blank determination performed under identical conditions. The endpoint was identified as a color change from green to golden yellow. The total tannin content was calculated using the standard indigo carmine titration equation and expressed as a percentage in dry weight: Tannins (%) = [(*V*S − *V*B) × 0.004157 × 250 × 100]/(*M* × 25), where *V*_S_ and *V*_B_ denote the sample and blank titer values, respectively; *M* is the sample mass in grams; 0.004157 is the tannin equivalent in 1 ml of 0.1 N aqueous solution of KMnO_4_; 250 is the volume of the volumetric flask (in milliliters); 100 is the percentage; and 25ml is the volume of the samples taken.

### 2.6 Biochemical analysis

The Folin–Ciocalteu method was used to measure the total phenolic content (TPC). Absorbance was measured at 750 nm, and the results are expressed as milligrams of gallic acid equivalent (GAE) per 100 g on a DW basis. The aluminum chloride colorimetric method was used to measure the total flavonoid content (TFC) (Ashraf et al., 2024; Radović et al., 2024). Absorbance was measured at 510 nm, and the results are expressed as milligrams quercetin equivalent (QE) per 100 g on a DW basis.

### 2.7 Antioxidant activity

The standard protocol was used to determine the free radical scavenging activity [2,2-diphenyl-1-picrylhydrazyl (DPPH) assay] (Abeysuriya et al., 2020; Cai et al., 2005). After 30 min incubation in the dark, absorbance was measured at 517 nm and the percentage of inhibition calculated. The 2,2'-azino-bis(3-ethylbenzothiazoline-6-sulfonic acid) (ABTS) radical scavenging activity (ABTS˙^+^) assay was used to determine the free radical scavenging capacity of the sample in accordance with the protocol (Ashraf et al., 2024). The results are expressed as micromoles Trolox equivalent (TE) per 100 g DW. Ferric reducing antioxidant power (FRAP) was used to determine the antioxidant capacity (Ashraf et al., 2024), which involves quantifying the ability of the sample to reduce ferric (Fe^3+^) ions to ferrous (Fe^2+^) ions. Absorbance was measured at 593 nm, and the results are expressed as micromoles Trolox equivalent (TE) per 100 g DW.


$$\text{Inhibition}\left(\%\right)=({\text{A}}_{\text{control}}-{\text{A}}_{\text{sample}})/{\text{A}}_{\text{control}}\times \;100$$

### 2.8 Structural property

#### 2.8.1 Fourier transform infrared spectroscopy

The intrinsic chemistry of the dragon fruit peel and the various functional elements were analyzed using IR Affinity-1, Thermo Nicolet iS50 Fourier transform infrared spectroscopy (Shimadzu, Japan, Thermo Fisher Scientific) in KBr mode, covering the spectral range from 4,000 to 500 cm^−1^ (Srinivasan and Rana, 2025).

#### 2.8.2 X-ray diffraction

The amorphous nature of the dragon fruit peel sample was examined using a Bruker D8 advanced X-ray diffractometer (Panalytical X’Pert3, Germany, Netherlands) with a diffraction angle range of 1°–80° (Kaur et al., 2021; Srinivasan and Rana, 2025).

#### 2.8.3 Scanning electron microscopy

The microstructural features and the surface morphology of the peel powder were assessed using a scanning electron microscope (CARL ZEISS EVO 18) equipped with an energy-dispersive X-ray spectroscopy (EDX) detector. The samples were attached to aluminum stubs using carbon adhesive tape before imaging by coating with a thin conductive platinum/gold layer to minimize the charging effects during use. The samples were photographed at various magnifications (Kaur et al., 2021; Srinivasan and Rana, 2025).

### 2.9 Statistical analysis

All experiments were conducted in triplicate, and the results are expressed as the mean ± standard deviation. Data were analyzed using two-way analysis of variance (ANOVA), with variety (*V*) and maturity stage (*M*) as the main factors and their interaction (*V* × *M*). When significant differences among maturity stages are detected, the treatment means within each variety were compared using Tukey’s honestly significant difference (HSD) test at the *p* < 0.01 significance level. Correlation analysis and hierarchical cluster analysis (HCA; Ward’s method) were also performed to assess relationships among the measured variables and the treatment groups. Statistical analyses were conducted using JMP Pro 18 Student Edition.

## 3 Results and discussion

### 3.1 Physical and quality attributes of dragon fruit peel

#### 3.1.1 Inedible index and morphological changes

**Figure 1:**
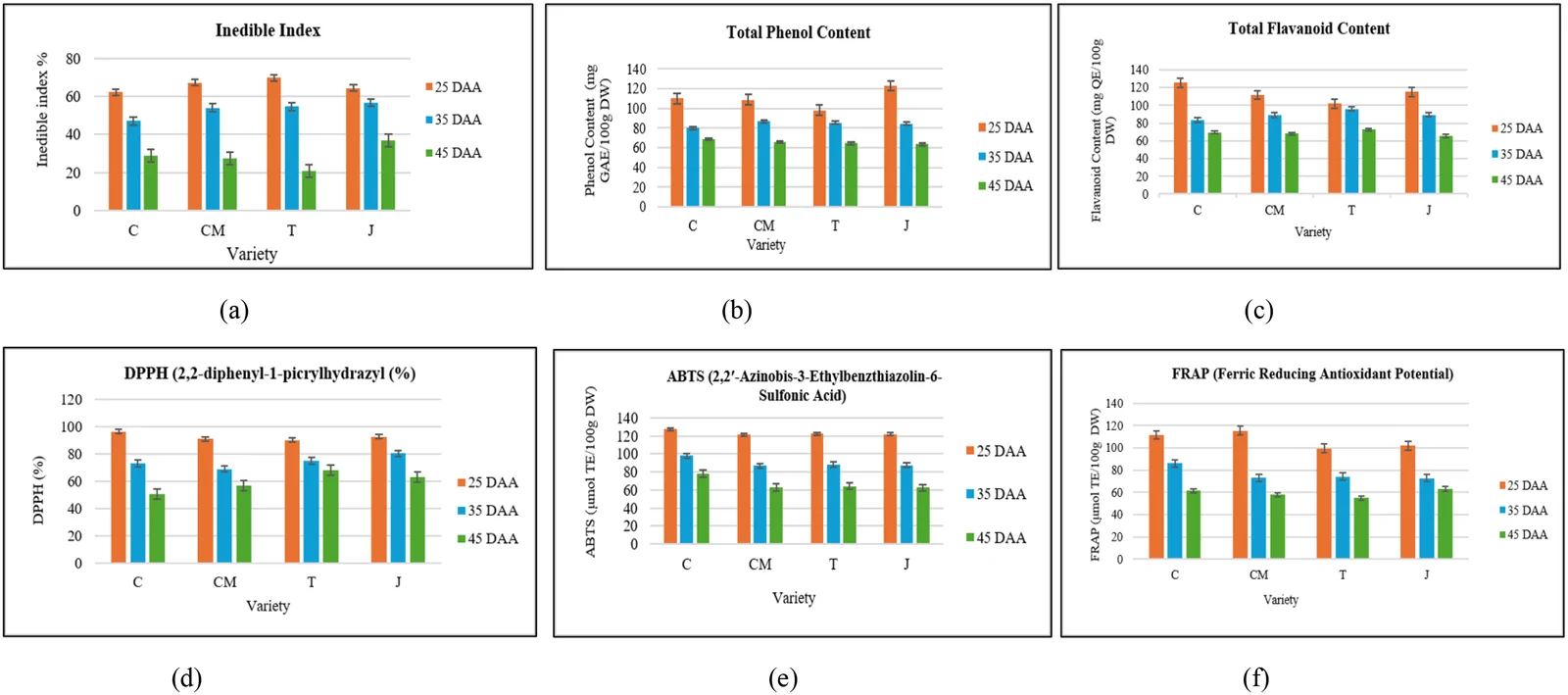
Bioactive and functional properties of dragon fruit peel from four *Selenicereus* spp. varieties (C, CM, T, and J) harvested at different maturity stages (25^th^, 35^th^, and 45^th^ DAA): **(a)** inedible index, **(b)** total phenol content, **(c)** total flavonoid content, **(d)** DPPH radical scavenging activity, **(e)** ABTS radical scavenging activity, and **(f)** FRAP antioxidant activity. Values represent mean ± SD (n = 3).

The peel mass relative to the total fruit mass was expressed as the inedible index, which decreased with maturity in all varieties (**Table 1**, **Figure 1**). The inedible index was calculated on a fresh weight basis as the percentage of peel mass (seeds excluded) relative to the total fruit weight. Seeds were retained with the pulp fraction during weighing and were therefore included in the edible/pulp portion rather than the peel fraction. The inedible index ranged from 62.29% to 69.80% at 25 DAA and declined to 20.96%–37.03% at 45 DAA. At full maturity, variety T recorded the numerically lowest inedible index (20.96%), whereas variety J showed the highest value (37.03%).

**Table 1:** Physical and biochemical parameters of the peel of four *Selenicereus* spp. varieties (C, CM, T, and J) evaluated on the 25th, 35th, and 45th day after anthesis (DAA).

Variable	Variety	Maturity			Variable	Variety	Maturity		
		25th DAA	35th DAA	45th DAA			25th DAA	35th DAA	45th DAA
Inedible index (%)	C	62.29 ± 0.10c	47.17 ± 2.36b	29.06 ± 3.77a	Ash (%)	C	1.97 ± 0.52a	6.0 ± 1.2b	10.06 ± 0.5c
	CM	67.18 ± 0.35c	54.01 ± 0.97b	27.44 ± 0.27a		CM	6.06 ± 0.55a	8.23 ± 0.61b	10.77 ± 0.64c
	T	69.80 ± 0.58c	53.65 ± 1.02b	20.96 ± 0.63a		T	1.96 ± 0.59a	4.74 ± 0.58b	6.7 ± 0.36c
	J	64.49 ± 1.79c	56.72 ± 0.59b	37.03 ± 2.0a		J	2.35 ± 0.04a	5.59 ± 0.05b	10.62 ± 0.58c
Color (L*)	C	19.3 ± 0.078a	27.7 ± 0.58b	33.6 ± 1.15c	Protein (%)	C	2.34 ± 0.21a	3.15 ± 0.04b	5.93 ± 0.62c
	CM	26.53 ± 1.2a	33.03 ± 0.5b	36.15 ± 0.12c		CM	1.04 ± 0.54a	4.29 ± 0.62b	5.55 ± 0.59c
	T	26.86 ± 1.00a	33.23 ± 0.61b	36.40 ± 0.71c		T	0.45 ± 0.03a	2.2 ± 0.55b	4.49 ± 0.59c
	J	26.6 ± 1.12a	33.16 ± 0.40b	36.12 ± 0.85c		J	0.39 ± 0.06a	2.26 ± 0.56b	4.56 ± 0.65c
a*	C	5.20 ± 0.12a	6.93 ± 2.0b	12.8 ± 0.54c	Fat (%)	C	1.11 ± 0.12a	3.06 ± 0.51b	5.403 ± 0.22c
	CM	9.28 ± 0.06a	11.96 ± 0.66b	14.07 ± 0.13c		CM	0.79 ± 0.25a	4.57 ± 0.58b	5.31 ± 0.20c
	T	9.29 ± 0.167a	12.1 ± 0.3b	14.13 ± 0.20c		T	0.126 ± 0.01a	3.30 ± 0.61b	5.43 ± 0.51c
	J	9.25 ± 0.09a	11.93 ± 0.47b	14.07 ± 0.12c		J	0.416 ± 0.01a	2.21 ± 1.13b	4.30 ± 0.56c
b*	C	3.4 ± 0.17c	1.51 ± 0.17b	0.69 ± 0.02a	Fiber (%)	C	12.78 ± 1.09a	19.56 ± 0.56b	24.61 ± 1.03c
	CM	3.25 ± 0.06c	1.43 ± 0.19b	0.61 ± 0.12a		CM	9.86 ± 0.55a	17.3 ± 0.20b	23.79 ± 0.82c
	T	3.2 ± 0.08c	1.49 ± 0.11b	0.633 ± 0.11a		T	9.81 ± 0.60a	15.63 ± 2.04b	20.97 ± 0.57c
	J	3.25 ± 0.09c	1.42 ± 0.47b	0.643 ± 0.11a		J	5.72 ± 0.05a	15.24 ± 0.60b	23.38 ± 1.30c
pH	C	4.1 ± 0.05b	4.07 ± 0.11a	4.78 ± 0.31c	Carbohydrates (%)	C	18.2 ± 0.25a	33.01 ± 0.58b	41.5 ± 1.16c
	CM	4 ± 0.11a	4.4 ± 0.05b	4.3 ± 0.15c		CM	19.31 ± 0.72a	33.83 ± 0.71b	41.13 ± 0.98c
	T	4.1 ± 0.31a	4.43 ± 0.05b	4.3 ± 0.01c		T	19.13 ± 1.0a	24.86 ± 0.74b	45.70 ± 0.68c
	J	3.96 ± 0.15a	4.46 ± 0.12b	4.3 ± 0.13c		J	9.55 ± 0.70a	22.76 ± 0.51b	43.61 ± 0.62c
Oil absorption capacity (g/g)	C	1.33 ± 0.05a	2.67 ± 0.89b	3.84 ± 0.06c	Energy (kcal/100 g)	C	92.52 ± 2.2a	172.22 ± 1.13b	247.71 ± 6.7c
	CM	1.65 ± 0.05b	1.42 ± 0.03a	2.66 ± 0.08c		CM	88.55 ± 6.20a	193.7 ± 9.61b	234.55 ± 3.94c
	T	1.66 ± 0.04b	1.48 ± 0.03a	3.97 ± 0.25c		T	87.47 ± 3.90a	120.59 ± 0.12b	215.21 ± 0.60c
	J	1.66 ± 0.04b	1.42 ± 0.04a	2.69 ± 0.07c		J	43.53 ± 2.46a	119.9 ± 11.9b	232.4 ± 9.4c
Water holding capacity (g/ml)	C	1.13 ± 0.10a	2.14 ± 0.01b	2.31 ± 0.90c	Moisture (%)	C	5.01 ± 0.80a	8.50 ± 0.55b	9.31 ± 0.43c
	CM	1.12 ± 0.01a	1.8 ± 0.07b	2.05 ± 0.05c		CM	3.92 ± 0.57a	3.88 ± 0.68a	8.17 ± 0.06b
	T	1.12 ± 0.02a	1.81 ± 0.05b	2.08 ± 0.06c		T	3.85 ± 0.74a	8.57 ± 0.34b	11.08 ± 0.72c
	J	1.12 ± 0.02a	1.79 ± 0.06b	2.06 ± 0.03c		J	2.45 ± 0.43a	4.92 ± 0.61b	8.33 ± 0.48c
TPC (mg GAE/100 g DW)	C	110.10 ± 0.28c	80.08 ± 0.75b	69.06 ± 0.49a	Swelling capacity (g/g)	C	4.19 ± 0.01a	5.15 ± 0.05b	6.27 ± 0.01c
	CM	109 ± 0.23c	86.95 ± 0.73b	65.70 ± 0.76a		CM	3.45 ± 0.18a	3.86 ± 0.25b	6.20 ± 0.25c
	T	98.30 ± 0.34c	85.44 ± 0.78b	64.60 ± 3.9a		T	3.51 ± 0.12a	3.86 ± 0.13b	6.32 ± 0.14c
	J	122.99 ± 0.49c	84.07 ± 1.60b	63.57 ± 1.62a		J	3.43 ± 0.12a	3.88 ± 0.25b	6.21 ± 0.29c
TFC (mg QE/100 g DW)	C	125.41 ± 3.76c	83.39 ± 3.2b	69.57 ± 5.7a	DPPH (%)	C	96.65 ± 0.10c	73.11 ± 0.37b	51.05 ± 0.66a
	CM	111.98 ± 0.56c	89.01 ± 0.61b	68.2 ± 1.79a		CM	91.19 ± 0.484c	69.11 ± 0.29b	57.15 ± 0.55a
	T	101.82 ± 0.77c	96.11 ± 0.85b	72.64 ± 0.91a		T	90.38 ± 1.05c	75.36 ± 1.11b	68.29 ± 0.97a
	J	115.16 ± 0.42c	89.31 ± 5.35b	65.5 ± 0.48a		J	92.96 ± 1.32c	80.53 ± 0.62b	63.2 ± 1.14a
FRAP (μmol TE/100 g DW)	C	111.71 ± 0.55c	86.23 ± 1.7b	61.49 ± 1.56a	ABTS (μmol TE/100 g DW)	C	127.47 ± 1.87c	97.92 ± 1.5b	78.19 ± 0.89a
	CM	115.5 ± 1.51c	73.15 ± 0.98b	57.78 ± 2.17a		CM	121.46 ± 0.80c	86.94 ± 1.35b	62.95 ± 1.51a
	T	99.6 ± 1.33c	74.07 ± 0.72b	55.14 ± 1.88a		T	122.2 ± 1.26c	88.28 ± 1.11b	64.15 ± 1.28a
	J	101.9 ± 1.15c	72.7 ± 0.82b	63.17 ± 1.88a		J	121.8 ± 0.95c	87.6 ± 1.26b	62.41 ± 1.30a

Values are expressed as the mean ± standard deviation (*n* = 3). Means within the same row followed by different lowercase letters are significantly different among maturity stages within the same variety according to Tukey’s honestly significant difference (HSD) test (*p* < 0.01).

*CM*, CM Pink; *T*, Taiwan Pink; *J*, Jumbo Red; *DW*, dry weight; *TPC*, total phenolic content; *GAE*, gallic acid equivalent; *TFC*, total flavonoid content; *QE*, quercetin equivalent; *FRAP*, ferric reducing antioxidant power; *TE*, Trolox equivalent.

The decline in the inedible index represents a decrease in the relative proportion of peel with respect to the total fruit weight (fresh weight basis) rather than a reduction in the absolute peel biomass. As fruit develops, pulp growth outpaces peel growth, resulting in a progressive reduction in the percentage contribution of peel to the total fruit weight. During fruit development, pulp parenchyma cells undergo extensive enlargement accompanied by vacuolar water uptake and soluble sugar accumulation, resulting in a substantially greater increase in pulp mass. In contrast, peel growth is comparatively slow and is primarily associated with structural reinforcement, including cell wall thickening and cuticle development. Consequently, the relative contribution of the peel to the total fruit weight decreases with maturity because pulp development progresses more rapidly than peel development. Similar trends have been reported in *Hylocereus* spp., where rapid pulp expansion during the later stages of fruit development reduces the proportion of peel despite continued peel development (Magalhães et al., 2019; Zitha et al., 2022). The varietal differences observed may be attributed to varietal variations in biomass partitioning and fruit development. Varieties C, CM, and T showed lower inedible indices at maturity, which may translate to higher edible yield and greater suitability for fresh consumption and processing, while the comparatively higher peel proportion of variety J may make it more advantageous for peel valorization targeting dietary fiber, minerals, and pigments. There are differences in the proportion of peel among the varieties, which can be explained by variety-dependent differences in biomass allocation, tissue development, and maturation behavior. Similar maturity-dependent changes in the peel proportion have been reported in dragon fruit and other fleshy fruits (Magalhães et al., 2019; Zitha et al., 2022).

#### 3.1.2 Color development during maturation

There was a constant change in the peel color during early and full maturity (**Table 1**). The L* values for the evaluated samples increased from 19.3 to 33.6 (variety C) and from 26.86 to 36.40 (variety J) during maturation, which reflects lightening of the surface. The a* value (red–green axis) ranged from 5.20–9.29 on the 25th DAA to 12.8–14.13 on the 45th DAA, indicating a stronger red tint. The b* value (yellow–blue axis) decreased and ranged between 3.2–3.4 on the 25th DAA and 0.61–0.69 on the 45th DAA. Among the varieties, the largest numerical change in the color parameters was observed in variety C.

These color dynamics indicate the breakdown of chlorophyll along with the production of betalains (betacyanin and betaxanthin) that remobilize the tyrosine-derived pathways in Caryophyllales, which are the main photochemical pigments in dragon fruit (Trong et al., 2022). It rises abruptly in the mid-to-late stages, coinciding with the phases reported to accumulate betalains (25th–32th DAA) in varieties with red coloration (Zitha et al., 2022). Therefore, color change is a reliable, noninvasive maturity indicator, and increases in a* and L* combined with decreases in b* correlate strongly with biochemical maturation and consumer-visible ripeness (Arivalagan et al., 2021).

#### 3.1.3 pH stability during maturation

The peel pH increased significantly with maturity, ranging from 3.9–4.1 on the 25th DAA to 4.3–4.73 on the 45th DAA across all varieties (**Table 1**). The small change in pH indicates that the organic acid metabolism in peel tissue remains relatively constant compared with that in the pulp, which tends to show a pH increase associated with respiration- and sugar-driven acid consumption during ripening. Maintaining the acidic peel pH can confer antimicrobial and antioxidant benefits, reduce the risk of microbial colonization, and help prevent enzymatic browning in subsequent developmental stages. This concentration difference in pH shift in variety C compared with the other varieties can be a result of varying organic acid profiles or a specific distribution of the metabolic activity, which can be further profiled on specific organic acids (e.g., citric, malic, and oxalic) to identify the biochemical drivers behind it (Trong et al., 2022; Zitha et al., 2022).

### 3.2 Functional properties

The OAC, WHC, and SC of the peel powder obtained under standard drying conditions are depicted in **Table 1**. The three functional traits increased with maturity (*p* < 0.01).

OAC increased from 1.33–1.66 g/g (25th DAA) to 2.66–3.97 g/g (45th DAA), and variety T had the highest value on the 45th DAA (3.97 g/g). A high OAC is associated with protein and hydrophobic polysaccharides, which facilitate lipid entrapment through capillary and hydrophobic interactions in plant-derived dietary fibers (Singh et al., 2020). The measured mature peel OAC values were comparable to several reported plant fiber ingredients, indicating that they can be used in fat retention applications.

The WHC in the given samples ranged from 1.12–1.13 g/ml (25th DAA) to 2.05–2.31 g/ml (45th DAA). Variety C recorded the highest WHC value (2.31 g/ml) on the 45th DAA. The WHC of peel indicates the ability of the polymeric polysaccharides and proteins present in the peel to form hydrogen bonds with water and reflects the content and porosity of soluble fibers, such as pectin and hemicelluloses. A high WHC is beneficial in high-moisture food systems as it helps sustain texture, extend shelf life (through moisture control), and aid food formulations.

SC increased from 3.43–4.19 g/g (25th DAA) to 6.20–6.32 g/g (45th DAA) across all varieties, with only minor varietal differences observed at full maturity. SC is highly sensitive to soluble fiber swelling (pectin) and plays a vital role in viscosity, gelation, and bulking in low-calorie formulations. The consistent results of >6 ml/g SC on the 45th DAA indicated the positive gelling and thickening ability of the peel. The totality of the maturity-dependent improvements in OAC, WHC, and SC indicates that underlying changes in composition will improve the dietary fiber (including soluble components), protein deposition, microstructural rearrangement, and the augmentation of binding sites. Mature peel can be used as a versatile hydrocolloid/fiber texture ingredient and as a water/oil management and dietary fiber fortification ingredient.

The improvements in WHC, OAC, and SC observed with maturity indicate an enhanced physicochemical functionality of dragon fruit peel. However, these properties do not directly reflect nutrient digestibility and bioefficacy, which were not evaluated in the present study. Therefore, further investigations using *in vitro* digestion and bioavailability are required.

The higher WHC, SC, and OAC observed among varieties may be associated with differences in the dietary fiber composition, cell wall polysaccharide organization, and microstructural characteristics. Genotypic variations in the relative proportions of cellulose, hemicellulose, pectin, and other structural polysaccharides may influence water binding, matrix expansion, and oil retention, thereby contributing to differences in the functional properties among varieties. In addition, variations in tissue development, cell wall, porosity, and surface characteristics may further influence hydration and swelling behavior. These observations suggest that variety-dependent compositional and structural differences contribute to the functional performance of dragon fruit peel powder and may influence the suitability of specific varieties for different food ingredient applications.

### 3.3 Proximate composition of dried peel powder

Proximate analysis of the peel samples indicated significant changes during maturation (**Table 1**). The moisture content increased marginally with maturity, ranging from 2.45% to 5.01% on the 25th DAA and from 8.17% to 11.08% on the 45th DAA. The ash levels increased significantly (*p* < 0.01) at full maturity, ranging from 6.7% to 10.77%, indicating that the concentrations of minerals in the peel tissue increased as it matured. An increase in protein content was observed during maturation. The protein content in the peel ranged from 0.39% to 2.34% on the 25th DAA and from 4.49% to 5.93% on the 45th DAA. Similarly, fat on the 25th DAA ranged from 0.13% to 1.11% and on the 45th DAA from 4.30% to 5.43%, which can be attributed to the deposition of cuticular wax and a buildup of membrane lipids in mature peel (contributing to higher OAC). Dietary fiber increased significantly, ranging from 5.72% to 12.78% on the 25th DAA and from 20.97% to 24.61% on the 45th DAA. The carbohydrate and nutrient energy levels also increased, with carbohydrates ranging from 41.13% to 45.70% on the 45^th^ DAA and energy from 215.21 to 247.71 kcal/100 g on the 45th DAA, the accumulation of which was supported by structural carbohydrates. These compositional changes are in line with the metabolic differences between a defensive, metabolically active tissue (abundant in soluble phenolics at the initial stages) and a structural, protective matrix (abundant in minerals, fibers, and cuticular lipids at the mature stages). This change aligns with trends in functional performance and with previous data on the dynamics of peel composition in dragon fruit and other fruits (Le, 2022; Zitha et al., 2022). The dietary fiber, protein, and mineral contents increased after the 35th DAA, along with increases in the WHC, OAC, and SC. These compositional and functional characteristics support the potential of mature dragon fruit peel. However, additional studies on nutrient bioavailability, processing, safety limits, and product formulation are required before its suitability for industrial applications can be determined.

### 3.4 Mineral composition

Analysis of the mineral composition revealed that the natural enrichment of the macro- and micro-minerals mostly occurred in the dragon fruit peel during the maturation period of 25–45 DAA (*p* < 0.01) (**Table 2**). The macro-mineral composition on the 25th DAA varied across varieties, as follows: calcium, 12.08–19.04 mg/100 g; magnesium, 17.64–19.66 mg/100 g DW; potassium, 11.12–22.04 mg/100 g DW; sodium, 18.02–33.04 mg/100 g DW; phosphorus, 19.04–36.06 mg/100 g DW; iron, 0.53–0.72 mg/100 g DW; manganese, 0.24–0.50 mg/100 g DW; and zinc, 1.04–2.96 mg/100 g DW. On the 45th DAA, these values increased: calcium, 47.04–52.02 mg/100 g DW; magnesium, 27.04–28.10 mg/100 g DW; potassium, 28.12–30.46 mg/100 g DW; sodium, 56.04–71.56 mg/100 g DW; phosphorus, 23.72–58.18 mg/100 g DW; iron, 1.82–1.96 mg/100 g DW; manganese, 1.04–1.30 mg/100 g DW; and zinc 2.10–2.72 mg/100 g DW. Among the varieties, variety T recorded the highest phosphorus concentration (58.18 mg/100 g DW). In contrast, varieties C and J recorded the highest potassium concentration (30.46 and 30.22 mg/100 g DW respectively), possibly due to variety-specific mineral transport processes (Ranty et al., 2016). The micro-minerals exhibited similar patterns, and the iron content increased across all varieties, reaching 1.82–1.96 mg/100 g DW on the 45th DAA, with iron accumulation being numerically highest in variety T. Zinc remained steady in variety CM (2.25–3.16 mg/100 g DW), and variety C recorded the highest manganese concentration (1.30 mg/100 g DW). Copper ranged from 0.34 to 1.10 mg/100 g DW, while the cobalt content remained low (0.04–0.41 mg/100 g DW) across all stages. The minerals observed are transported through the xylem and phloem, deposited in the vacuoles, and attached to the cell wall matrix (Martínez-Rivas and Fernie, 2024). High calcium aids pectin cross-linking to increase peel strength, while potassium maintains fruit turgor (Leterme et al., 2006). The differences in the mineral accumulation among varieties could be related to differences in the nutrient uptake efficiency, the xylem and phloem transport capacity, sink strength, and storage in peel tissues. Varietal variations in the nutrient concentrations and developmental physiology may therefore contribute to the observed variations in the mineral composition. Dragon fruit peel on the 45th DAA showed potential as an appreciable source for food fortification, with varieties suitable for specific applications (Zitha et al., 2022; Arivalagan et al., 2021; Lalduhsangi and Mandal, 2023; Trong et al., 2022).

**Table 2:** Mineral composition (Ca, Fe, K, Mg, Mn, Na, P, and Zn) of dried fruit peel powder from four *Selenicereus* spp. varieties across three (25th, 35th, and 45th day after anthesis, DAA) maturity stages.

Variable	Variety	Maturity stage		
		25th DAA	35th DAA	45th DAA
Calcium (Ca) (mg/100 g DW)	C	18.10 ± 0.56a	38.06 ± 1.41b	49.12 ± 0.88c
	CM	19.04 ± 0.52a	48.02 ± 0.98b	51.00 ± 1.12c
	T	12.08 ± 1.28a	33.04 ± 1.18b	52.02 ± 1.02c
	J	14.02 ± 1.08a	45.00 ± 0.70b	47.04 ± 0.38c
Cobalt (Co) (mg/100 g DW)	C	0.11 ± 0.01a	0.30 ± 0.07c	0.25 ± 0.05b
	CM	0.40 ± 0.28c	0.28 ± 0.01b	0.15 ± 0.02a
	T	0.25 ± 0.06b	0.04 ± 0.01a	0.36 ± 0.10c
	J	0.04 ± 0.02a	0.09 ± 0.02b	0.12 ± 0.04c
Copper (Cu) (mg/100 g DW)	C	0.66 ± 0.10a	0.79 ± 0.00b	0.87 ± 0.14c
	CM	0.63 ± 0.02a	0.92 ± 0.09c	0.89 ± 0.07b
	T	0.71 ± 0.23a	0.37 ± 0.14b	0.69 ± 0.21c
	J	0.39 ± 0.08b	0.34 ± 0.03a	1.10 ± 0.49c
Iron (Fe) (mg/100 g DW)	C	0.53 ± 0.12a	0.94 ± 1.14b	1.88 ± 1.21c
	CM	0.53 ± 0.49a	0.88 ± 1.26b	1.89 ± 0.28c
	T	0.54 ± 0.07a	0.92 ± 0.82b	1.96 ± 1.04c
	J	0.72 ± 0.01a	0.68 ± 0.32b	1.82 ± 0.30c
Potassium (K) (mg/100 g DW)	C	22.04 ± 1.62a	29.08 ± 1.90b	30.46 ± 0.90c
	CM	11.12 ± 1.12a	21.02 ± 1.4b	29.16 ± 0.73c
	T	14.04 ± 0.38a	20.02 ± 0.39b	28.12 ± 0.72c
	J	18.10 ± 0.24a	22.04 ± 1.26b	30.22 ± 1.52c
Magnesium (Mg) (mg/100 g DW)	C	18.02 ± 0.24a	22.04 ± 1.62b	28.10 ± 0.18c
	CM	19.12 ± 0.52a	22.14 ± 1.38b	27.08 ± 0.20c
	T	19.66 ± 1.45a	21.08 ± 1.45b	27.72 ± 0.68c
	J	17.64 ± 1.39a	21.62 ± 1.33b	27.04 ± 0.84c
Manganese (Mn) (mg/100 g DW)	C	0.24 ± 0.18a	0.78 ± 0.07b	1.30 ± 0.19c
	CM	0.42 ± 0.20a	0.36 ± 0.11b	1.04 ± 0.26c
	T	0.34 ± 0.29a	0.56 ± 0.19b	1.18 ± 1.06c
	J	0.50 ± 0.33a	0.54 ± 0.26b	0.56 ± 0.40c
Sodium (Na) (mg/100 g DW)	C	32.04 ± 1.14a	36.08 ± 1.30b	68.48 ± 0.32c
	CM	33.02 ± 1.16a	43.09 ± 0.96b	71.56 ± 0.57c
	T	18.04 ± 0.47a	22.04 ± 0.30b	56.04 ± 1.42c
	J	23.08 ± 0.86a	36.04 ± 0.37b	58.68 ± 0.90c
Phosphorus (P) (mg/100g DW)	C	22.04 ± 0.32a	22.50 ± 0.20b	23.72 ± 0.94c
	CM	19.04 ± 1.04a	27.08 ± 0.82b	29.08 ± 0.16c
	T	36.06 ± 1.07a	52.04 ± 0.96b	58.18 ± 1.13c
	J	32.12 ± 0.90a	42.08 ± 0.88b	45.28 ± 0.80c
Zinc (Zn) (mg/100 g DW)	C	1.04 ± 0.06a	1.56 ± 0.06b	2.10 ± 0.93c
	CM	2.52 ± 0.50a	3.16 ± 0.09b	2.25 ± 0.62c
	T	2.96 ± 0.96b	1.06 ± 0.15b	2.72 ± 0.23c
	J	1.22 ± 0.57a	1.34 ± 0.10b	2.16 ± 0.03c

Values are expressed as the mean ± standard deviation (*n* = 3). Means within the same row followed by different lowercase letters are significantly different among maturity stages within the same variety according to Tukey’s honestly significant difference (HSD) test (*p* < 0.01).

*DW*, dry weight; *CM*, CM Pink; *T*, Taiwan Pink; *J*, Jumbo Red.

### 3.5 Bioactive compounds and antioxidant activity

#### 3.5.1 Total phenolic content

The TPC decreased significantly with maturity (**Table 1**, **Figure 1**), ranging from 98.30–122.99 mg GAE/100 g DW on the 25th DAA to 63.57–69.06 mg GAE/100 g DW on the 45th DAA. The highest TPC was recorded in variety J on the 25th DAA (122.99 mg GAE/100 g DW). The decline in phenolic content during maturation may be attributed to polyphenol oxidase (PPO)-mediated oxidation, polymerization, and the incorporation of phenolic compounds into insoluble cell wall matrices or lignin-like structures (Rebecca et al., 2010; Macheix et al., 2018; Mayer, 2006; Esquivel et al., 2007). In addition, the reduction in free phenolic compounds may be associated with maturity-related metabolic changes. Other studies have proposed that the phenolic precursors may play a role in betalain production during fruit ripening; however, there was no measurement of betalain content in the present study.

#### 3.5.2 Total flavonoid content

Similarly to TPC, the TFC decreased significantly with maturity (*p* < 0.01) (**Table 1**, **Figure 1**). TFC ranged from 101.82–125.41 mg QE/100 g DW on the 25th DAA and declined to 65.50–72.64 mg QE/100 g DW on the 45th DAA. Flavonoids are susceptible to oxidative degradation, glycosylation, and interactions with the cell wall components during fruit maturation, which may reduce their extractability (Magalhães et al., 2019). The higher initial TFC observed among varieties suggests variety-dependent regulation of the flavonoid biosynthesis pathways, potentially reflecting differences in enzyme activity and secondary metabolite accumulation during fruit development.

#### 3.5.3 Antioxidant assays

The antioxidant activity, which was determined with the DPPH, ABTS, and FRAP assays, showed a consistent decline with maturity across all varieties (**Table 1**, **Figure 1**). The DPPH radical scavenging activity decreased from 90.38%–96.65% inhibition on the 25th DAA to 51.05%–68.29% inhibition on the 45th DAA. Similarly, the ABTS values declined from 121.46–127.47 μmol TE/100 g DW on the 25th DAA to 62.41–78.19 μmol TE/100 g DW on the 45th DAA, while the FRAP values decreased from 99.60–115.50 μmol TE/100 g DW to 55.14–63.17 μmol TE/100 g DW over the same maturity period. The consistent decline observed across all three assays suggests a reduction in the electron-donating and electron-reducing compounds during fruit maturation rather than an artifact of a particular analytical method. The close association between TPC, TFC, and antioxidant activity indicates that phenolic and flavonoid compounds are major contributors to the antioxidant potential of dragon fruit peel, consistent with previous reports (Lalduhsangi and Mandal, 2023).

Varietal differences might be attributed to varietal differences in phenylpropanoid metabolism, antioxidant enzyme activity, and pigment biosynthesis pathways. These physiological differences could influence the synthesis, accumulation, and degradation of the phenolic compounds during fruit ripening, thereby leading to differences in antioxidant activity among varieties. The antioxidant activity was high during the early maturity stages; however, mature peels retained appreciable antioxidant activity while exhibiting significantly improved nutritional properties, such as dietary fiber, minerals, WHC, OAC, and SC. Thus, the suitability of mature peel is mainly attributed to its nutritional and technological properties rather than solely to its antioxidant properties. However, when antioxidants are desired, immature peels may be preferred. Evaluation of the antioxidant capacity using the DPPH, ABTS, and FRAP assays aids in understanding the antioxidant activity of dragon fruit peel extracts. Nevertheless, they do not reflect the digestion, absorption, metabolism, and tissue distribution of the antioxidants in biological systems. Therefore, the observed antioxidant activity is not evidence of *in vivo* activity or health benefits, but rather an indication of antioxidant potential. In addition, the low values for the phenolic content, flavonoids, and antioxidants during fruit maturation might be related to the oxidation of phenolic compounds and other metabolic changes that occur during this stage (Babaei Rad et al., 2025; Sagar et al., 2018). Previous studies have suggested that phenolic precursors may be diverted toward betalain biosynthesis during fruit ripening (Strack et al., 2003). However, betalain pigments were not quantified in the present study. Therefore, this explanation should be regarded as a possible mechanism based on previous literature rather than a confirmed process. Further studies, including quantification of betalains, are required to verify this relationship.

### 3.6 Anti-nutritional factors

#### 3.6.1 Phytic acid

The phytic acid content in all varieties showed a slow reduction trend between the 25th DAA (4.43–4.68 mg/100 g DW) and the 45th DAA (3.11–3.84 mg/100 g DW) maturity period, with the lowest levels of phytic acid recorded in variety J (3.11 mg/100 g DW) and variety C (3.36 mg/100 g DW) at full maturity (**Table 3**). This trend may be linked to the breakdown of phytase-mediated phytate and the release of phosphorus during tissue maturation. Similar decreases have been reported in citrus and wild edible fruits, in which phytate serves as a temporary reservoir of phosphorus (Ani and Abel, 2018; Islary et al., 2018; Ngurthankhumi et al., 2024). The relatively low phytic acid concentrations observed at full maturity suggest a limited potential to interfere with mineral availability compared with the levels commonly reported in plant-derived foods. However, the actual impact on mineral bioavailability requires confirmation through *in vitro* digestion and bioavailability studies.

**Table 3:** Anti-nutritional components in dried peel powder of four *Selenicereus* spp. varieties at three (25th, 35th, and 45th day after anthesis, DAA) maturity stages.

Variable	Variety	Maturity stage		
		25th DAA	35th DAA	45th DAA
Phytic acid (mg/100 g DW)	C	4.68 ± 0.64c	4.37 ± 1.25b	3.35 ± 0.20a
	CM	4.43 ± 1.15c	4.50 ± 0.75b	3.80 ± 0.05a
	T	4.59 ± 0.71c	4.36 ± 0.37b	3.84 ± 0.20a
	J	4.57 ± 0.07c	4.30 ± 0.45b	3.11 ± 0.90a
Oxalate (mg/100 g DW)	C	0.94 ± 0.32a	2.09 ± 0.03b	2.31 ± 0.22c
	CM	1.41 ± 0.31a	2.06 ± 0.32b	4.87 ± 0.31c
	T	0.75 ± 0.32a	2.25 ± 0.12b	3.19 ± 0.32c
	J	1.62 ± 0.32a	1.56 ± 0.27b	2.43 ± 0.13c
Alkaloids (mg/100 g DW)	C	0.05 ± 0.01a	0.06 ± 0.02b	0.55 ± 0.10c
	CM	0.04 ± 0.01a	0.05 ± 0.02b	0.58 ± 0.03c
	T	0.04 ± 0.05a	0.05 ± 0.03b	0.50 ± 0.01c
	J	0.05 ± 0.03a	0.05 ± 0.01b	0.53 ± 0.06c
Tannins (% DW)	C	4.02 ± 0.63a	3.60 ± 0.15b	7.76 ± 0.13c
	CM	2.64 ± 0.25a	2.91 ± 0.42b	5.13 ± 0.24c
	T	1.94 ± 0.31a	2.36 ± 0.34b	4.02 ± 0.04c
	J	3.19 ± 0.24a	2.08 ± 0.32b	4.44 ± 0.48c

Values are expressed as the mean ± standard deviation (*n* = 3). Means within the same row followed by different lowercase letters are significantly different among maturity stages within the same variety according to Tukey’s honestly significant difference (HSD) test (*p* < 0.01).

*DW*, dry weight; *CM*, CM Pink; *T*, Taiwan Pink; *J*, Jumbo Red.

#### 3.6.2 Oxalates

The level of oxalate accumulated with maturity, especially in the CM and T varieties, i.e., 4.87 and 3.19 mg/100 g DW on the 45th DAA, respectively. This is likely associated with the deposition of calcium oxalate crystals, which strengthen epidermal tissues and regulate intracellular calcium levels, as reported in various tropical fruits (Ani and Abel, 2018; Islary et al., 2018). Although these are higher than those in the pulp, they remain manageable when incorporated at realistic inclusion levels, particularly when peel is used as a fortifying component.

#### 3.6.3 Alkaloids

The alkaloid content was generally low and ranged 0.04–0.05 mg/100 g DW on the 25th DAA and 0.50–0.58 mg/100 g DW on the 45th DAA. The increase in variety CM was relatively modest at the later stages of maturity. These levels suggest that the antioxidant and defensive activities of alkaloids in the peel are residual rather than increasing to significant toxic levels. Similar behavior has been reported in cactus and wild fruit peels, where protective secondary metabolites are retained to a greater extent in peel tissues than in pulp tissues (Ngurthankhumi et al., 2024).

#### 3.6.4 Tannins

Tannins increased steadily with maturity, ranging from 1.94% DW (variety T) to 4.02% DW (C variety) on the 25th DAA and from 4.02% DW (variety T) to 7.76% DW (variety C) on the 45th DAA. The increase in tannin content may be associated with the polymerization and incorporation of phenolic compounds into the cell wall structures during fruit maturation, contributing to peel firmness, pigmentation, and oxidative stability (Ani and Abel, 2018). Phenolic profiling also indicated that dragon fruit peel contains higher concentrations of phenolic compounds than the pulp, which may explain the comparatively higher tannin levels observed in peel tissues (Chen et al., 2021). These findings suggest that dragon fruit peel undergoes coordinated biochemical changes during maturation, characterized by a reduction in phytic acid, increases in the oxalates and tannins associated with structural reinforcement and defense mechanisms, and the relatively low alkaloid concentrations. Similar trends have been reported in tropical and wild fruits, where peel tissues continue to maintain protective and structural functions during ripening (Islary et al., 2018; Ngurthankhumi et al., 2024; Adamczyk et al., 2017). Despite the increase in the anti-nutritional factors, the observed concentrations remained relatively low and did not negate the nutritional and functional advantages associated with mature peel. Therefore, mature dragon fruit peel powder may be considered an ingredient for food formulations. However, further studies on nutrient bioavailability, dietary exposure, and safety assessment are required to confirm its suitability for food applications.

The concentrations of oxalate and tannin generally increased with maturity, although the trend was not strictly monotonic in all varieties. Variety J showed a slight decline in oxalate, while varieties C and J showed a transient decline in tannin between the 25th and 35th DAA before increasing by the 45th DAA, whereas phytic acid decreased consistently across all varieties. Oxalates can decrease the bioavailability of minerals, particularly calcium, by forming insoluble oxalates that limit intestinal absorption (Salgado et al., 2023). Similarly, tannins may interact with proteins and minerals to form complexes that can reduce protein digestibility and affect nutrient bioavailability (Ozdal et al., 2013; Yoshimura et al., 2025). Although the present study quantified these anti-nutritional compounds, it did not evaluate nutrient bioavailability, dietary exposure, or toxicological effects. Therefore, the nutritional implications of these compositional changes cannot be determined solely from the present data. Further studies are required to evaluate the *in vitro* digestion and bioavailability.

The phytic acid, oxalate, and tannin contents of dragon fruit peel powder were compared with the values reported for other commonly consumed fruit peels, including apple (*Malus pumila*), sweet lime (*Citrus limetta*), banana (*Musa acuminata*), and papaya (*Carica papaya*) (Gupta et al., 2020). The phytic acid content of dragon fruit peel powder (3.11–4.68 mg/100 g DW) was lower than the values reported for apple (146.66 mg/100 g), sweet lime (251.66 mg/100 g), banana (7.27 mg/100 g), and papaya (5.50 mg/100 g) peels. Similarly, the oxalate content (0.75–4.87 mg/100 g DW) was lower than the reported values for apple (85.36 mg/100 g), sweet lime (55.60 mg/100 g), banana (247.00 mg/100 g), and papaya (43.42 mg/100 g) peels. The tannin content (1.94%–7.76% DW) was also lower than the reported values for apple (75.34%), sweet lime (67.28%), banana (42.30%), and papaya (54.33%) peels. Although these compounds are recognized as anti-nutritional factors as they may reduce the bioavailability of minerals and proteins through complex formation, their nutritional significance depends on the dietary intake, food matrix, processing, and nutrient bioavailability. As the present study did not evaluate dietary exposure, nutrient bioavailability, or toxicological effects, no definitive conclusions regarding the nutritional safety of dragon fruit peel powder can be drawn. Therefore, further studies involving dietary exposure assessment, bioavailability, and toxicological evaluation are required.

### 3.7 Correlation and hierarchical cluster analysis of peel characteristics

**Figure 2:**
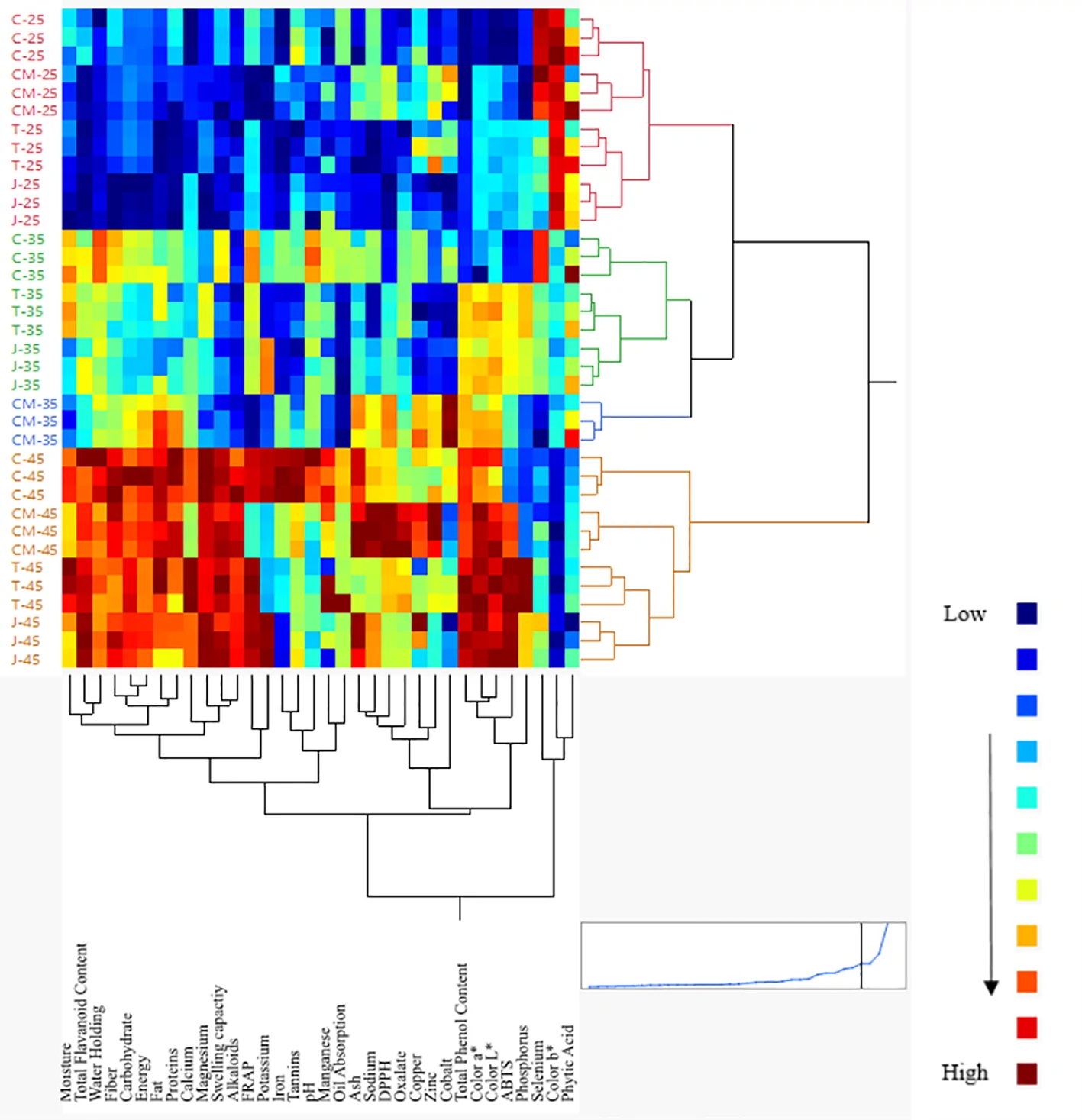
Hierarchical clustering heat-map of dragon fruit peel from four *Selenicereus* spp. varieties harvested at different maturity stages based on physicochemical, nutritional, antioxidant, mineral, and anti nutritional parameters.

Correlation analysis and hierarchical clustering were combined to enhance our understanding of the peel maturation process. While the correlation analysis explained the metabolic relationships among variables, hierarchical clustering revealed the collective effect across the different maturity stages (**Figure 2**). Hierarchical clustering with Spearman’s correlation analysis revealed maturity-induced biochemical reorganization in *Selenicereus* peel. The analysis assessed the biochemical relationships and multivariate similarities among the properties at three maturity levels (25th, 35th, and 45th DAA). This methodology provides system-level insights into peel maturation, beyond individual variables. The dendrogram grouped samples by maturity stage, indicating that the maturity stage is the primary predictor of peel composition. Peel from early-maturing samples showed lower carbohydrate, energy, color, and mineral contents, whereas fully matured samples had higher nutritional and functional attributes. These patterns align with the correlation findings, showing progressive metabolic changes during maturation. Early maturity showed positive relationships between TPC and TFC (*r* = 0.800) and among antioxidant measurements (DPPH and ABTS: *r* = 0.800; DPPH and FRAP: *r* = 0.600), indicating coordinated phenolic-based antioxidant metabolism in these cultivars. This finding is consistent with the HCA results and previous research on the antioxidant capacity of pitaya peel (Tang et al., 2021; Le, 2022). The energy-related variables and the antioxidant clusters showed negative correlations with carbohydrates, TPC (*r* = −0.800), and TFC (*r* = −0.600). Moisture content was positively correlated with the protein, fiber, and energy content (*r* = 0.996), indicating a strong association among the variables. This may be influenced by the limited data points and compositional interdependence, suggesting that hydration aids in structural and nutritional accumulation. On the 35th DAA, the phenolic–antioxidant relationships were reversed, with TPC showing a negative relationship with DPPH and ABTS (*r* = −0.800). This shift was also accompanied by some overlap among the antioxidant, fiber, tannin, and mineral clusters in the HCA, suggesting a redistribution of certain metabolic functions rather than a sudden biochemical loss. These changes have been attributed to the variations in pigment composition and the redistribution of bound phenolics during ripening (Ani and Abel, 2018; Priatni and Pradita, 2015; Tang et al., 2021).

The positive correlation between FRAP and ABTS (*r* = 0.800) indicates that the decreasing power and radical scavenging assays intercept the antioxidant processes at mid-maturity. The perfect correlations between carbohydrates, energy, and phytic acid (*r* = 0.996) indicate the simultaneous accumulation of energy reserves and phosphate storage in seeds. The clustering and correlation analyses revealed that the maturity stage is a metabolic transition between defense, structure, and energy deposition, which aligns with pitaya fruit ripening. A negative TPC–DPPH correlation indicates total phenolics decoupling from radical scavenging, while TPC–ABTS remained positive (*r* = 0.800), indicating some phenolic contribution to antioxidant mechanisms. The antioxidant activity aligned with the color parameters and minerals, suggesting a shift to non-phenolic antioxidants, likely betalain pigments, in mature dragon fruit peel. Minerals clustered with fibers and flavonoids, showing positive associations between TFC and Ca, Fe, and Mg at maturity (*r* = 0.800–0.996), indicating mineral–polyphenol complexation. Tannins were grouped with protein, fiber, and energy, showing protein–polyphenol reactions that formed peel rigidity. Phytic acid forms complexes with carbohydrates and minerals as phosphate storage compounds. While these compounds potentially reduce mineral bioavailability, they enhance peel stability when combined with other structural components.

WHC, OAC, and SC were grouped with moisture, fiber, and protein at full maturity. The correlation analysis showed that the moisture, protein, and tannin levels were positively correlated with WHC in early maturity, whereas OAC changed from a negative correlation with moisture and fiber on the 25th DAA to a positive correlation with moisture on the 45th DAA (*r* ≈ 0.995). These trends indicate major structural changes during ripening, which align with the reported changes in polysaccharides and fibers in dragon fruit peel (Tang et al., 2021). The correlation between OAC and the antioxidant parameters at mid-maturity suggests the potential of mature peel as a multifunctional component with lipid-binding and antioxidant properties. The correlation analysis revealed that *Selenicereus* peel underwent gradual metabolic reorganization based on maturity. Early maturity features phenolic-induced antioxidants, while mid-maturity balances structural and bioactive features. These findings indicate that harvest maturity should be determined based on the intended use. Early maturity (25th DAA) was optimal for phenolic antioxidant extraction, whereas full maturity (45th DAA) provided the best nutritional density and functionality for a value-added product.

### 3.8 Structural characterization and structure–functional relationship

The structural changes revealed by the scanning electron microscopy (SEM), Fourier transform infrared spectroscopy (FTIR), and X-ray diffraction (XRD) analyses may contribute to the improved functional properties of mature dragon fruit peel powder. The SEM micrographs showed that porosity increased with maturity, and the compact cellular structures became increasingly disrupted, creating more void space to hold water and retain oil. The observed increases in WHC, OAC, and SC are consistent with these structural changes. The FTIR spectra confirmed the presence of hydroxyl-rich polysaccharide components associated with dietary fiber. These hydrophilic functional groups may facilitate hydrogen bond interactions with water molecules, which increase hydration and swelling behavior. Although the XRD analysis indicated increased cellulose crystallinity at full maturity, the hydration property is influenced by multiple structural factors, including porosity, dietary fiber, and the balance of crystalline and amorphous regions. Therefore, the improved WHC and SC observed in mature peels may have resulted from the combined effects of the compositional and structural changes during fruit maturation rather than cellulose crystallinity.

#### 3.8.1 Scanning electron microscopy

**Figure 3:**
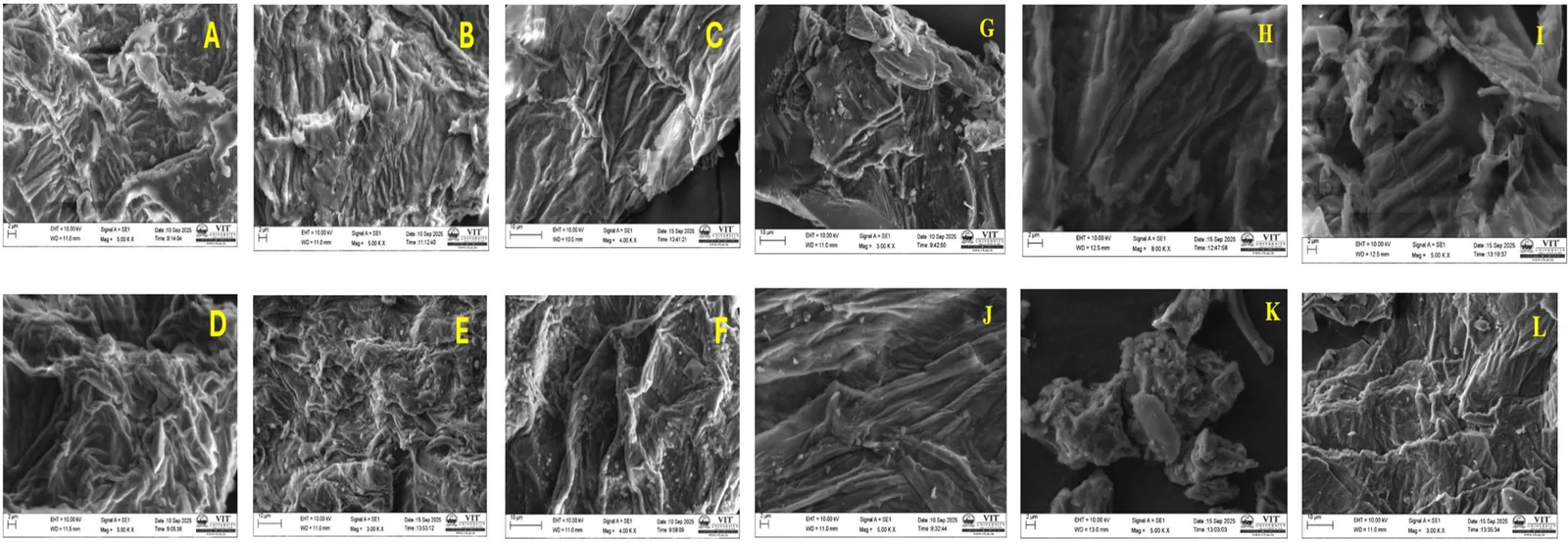
Scanning electron microscopy (SEM) images of dragon fruit peel from four varieties *Selenicereus* spp (C, CM, T, and J) harvested at 25^th^, 35^th^, and 45^th^ DAA. **(A–C)** C (25^th^, 35^th^, and 45^th^ DAA); **(D–F)** CM (25th, 35th, and 45th DAA); **(G–I)** T (25^th^, 35^th^, and 45^th^ DAA); **(J–L)** J (25^th^, 35^th^, and 45^th^ DAA). Micrographs demonstrate maturity-associated alterations in peel microstructure, characterized by variations in surface topology, fiber organization, and matrix integrity. Scale bars are provided in each micrograph.

The peel surface morphology of four different dragon fruit varieties (C, CM, T, and J) at three maturity stages (25th, 35th, and 45th DAA) was assessed using SEM (**Figure 3**). Structural differences were observed, implying the progressive organization of tissues as they matured. Variety C on the 25th DAA (**Figure 3A**) had a rough, highly porous surface and flake-like structures with fragmented, visible fissures, indicating a loosely assembled cellular matrix. These irregular morphological features have been replicated in dragon fruit peel pectin, which has granular nodules due to dispersed molecular components (Zhang et al., 2024). A gradual cell wall was observed after 35th DAA (**Figure 3B**), with increased structural organization of fiber bundles and decreased fragmentation. The peel at full maturity (**Figure 3C**) exhibited a well-compact fibrous structure with reduced porosity, indicating structural stabilization. The CM variety exhibited a highly interwoven fibrous network after 25th DAA, appearing as a mat with interconnected pores (**Figure 3D**). Reduced pore spaces and parallel-oriented bundles, reflecting increased structural rigidity, were observed in the fibers on the 35th DAA (**Figure 3E**).

In contrast, an increased pore space was observed on the 45th DAA (**Figure 3F**). Similar fiber structures in the form of needles have been reported in dragon fruit peel dietary fiber after thermal processing (Srinivasan and Rana, 2025). The T and J varieties had a relatively well-organized morphology at early maturity, with striated surfaces and parallel grooves on the 25th DAA (**Figures 3G, J**). The surface smoothness and fiber alignment improved on the 35th DAA (**Figures 3H, K**). The completely developed peel on the 45th DAA (**Figures 3I, L**) exhibited tightly packed bundles with a few voids, indicating a highly consolidated structure. The results are consistent with continuous and crackle morphologies, with enhanced structural integrity in pectin films (Zhang et al., 2024). The SEM images showed maturity-related changes, from irregular, porous structures to compact, organized fiber networks. The development is accompanied by an increase in crystallinity, indicating the deposition of cellulose and the rearrangement of polysaccharides during fruit development. This type of morphological development has been associated with functional characteristics such as hydration behavior and mechanical strength (Srinivasan and Rana, 2025). These microstructural changes are found to be maturity-dependent, which is consistent with the increases in WHC, OAC, and SC, indicating that the functional performance of dragon fruit peel powder is related to porosity and fiber network organization.

#### 3.8.2 Fourier transform infrared

**Figure 4:**
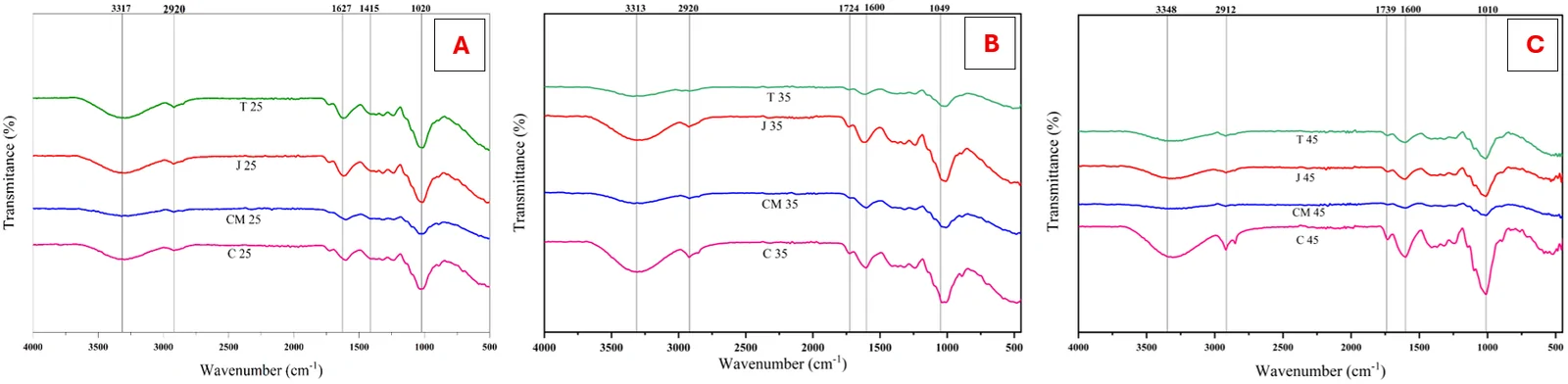
Fourier-transform infrared (FTIR) spectra of dragon fruit peel from four varieties *Selenicereus* spp (C, CM, T, and J) harvested at 25^th^, 35^th^, and 45^th^ DAA. **(A–C)** Overlaid spectra comparing all varieties at each maturity stage (A- 25^th^, B-35^th^, and C- 45^th^ DAA), demonstrating stage-dependent variations in functional group profiles.

To assess the influence of maturity, functional group changes in dragon fruit peel at various stages of maturity were evaluated using FTIR spectroscopy (**Figure 4**). Functional group absorption bands were observed in all samples: the chemical composition varied, with a predominant presence of polysaccharides and phenolic components. The presence of a broad band at 3,313–3,332 cm^−1^ is linked to the O–H stretching bands related to the hydroxyl groups of the polysaccharides and phenolic compounds (Srinivasan and Rana, 2025). The intensity of this band was higher in the 25th DAA samples and decreased with maturity, suggesting that the hydroxyl-rich structures were modified during ripening. The absorption peak at 2,916–2,931 cm^−1^, corresponding to the aliphatic cellulose and structural carbohydrate C–H stretch, also intensified as the peel reached the advanced maturity stage, indicating greater maturity of the structural components in the peel matrix. Bands were also observed at 1,600–1,616 cm^−1^, corresponding to carbonyl and aromatic C=C vibrations, characteristic of pectic substances (Hanan Taharuddin et al., 2023). An increase in intensity at early maturity and a decrease in intensity in the late stages indicated a reduction in the phenolic- and pectin-related functional groups during fruit development. The peaks in the 1,000- to 1,030-cm^−1^ domain correspond to C–O and C–O–C vibrations of cellulose, hemicellulose, and pectin. The increased broadening of the peaks and the decrease in strength during maturity evidenced the structural rearrangement of cell wall polysaccharides. Considering the impact of varietal differences on the functional characteristics at early maturity, variety C showed stronger hydroxyl and polysaccharide bands.

In contrast, at advanced maturity, varieties CM and J showed higher attenuation bands. The strongest spectral transitions were recorded for variety T, indicating that this peel exhibited faster biochemical alterations during peel maturation. In general, the FTIR data showed a higher proportion of hydroxyl-, aromatic-, and polysaccharide-related functional groups in immature peels, suggesting an aliphatic nature and polysaccharide rearrangement during maturation. These results indicate that fruit maturity is the major determinant of functional group growth in dragon fruit peels. In addition to the changes related to maturity, varietal differences also contributed to distinct FTIR spectral characteristics in dragon fruit peels. At all maturity levels, varieties C, CM, T, and J showed differences in peak intensity and band sharpness, particularly in the regions associated with polysaccharides, phenolics, and cell wall constituents. These variations indicate varietal differences in the biochemical content and the structural arrangement of peel tissues. The larger peels or those with greater fiber content showed stronger absorptions in carbohydrate-associated areas, whereas others showed mostly strong bands associated with phenolics and organic acids. Therefore, although the development of functional groups is largely controlled by fruit maturity, the level of variation and the degree of expression of these molecular adjustments are influenced by varietal traits. These microstructural changes are found to be maturity-dependent, which is consistent with the observed improvements in WHC, OAC, and SC, suggesting that changes in porosity and fiber network organization may contribute to functional performance.

#### 3.8.3 X-ray diffraction analysis

**Figure 5:**
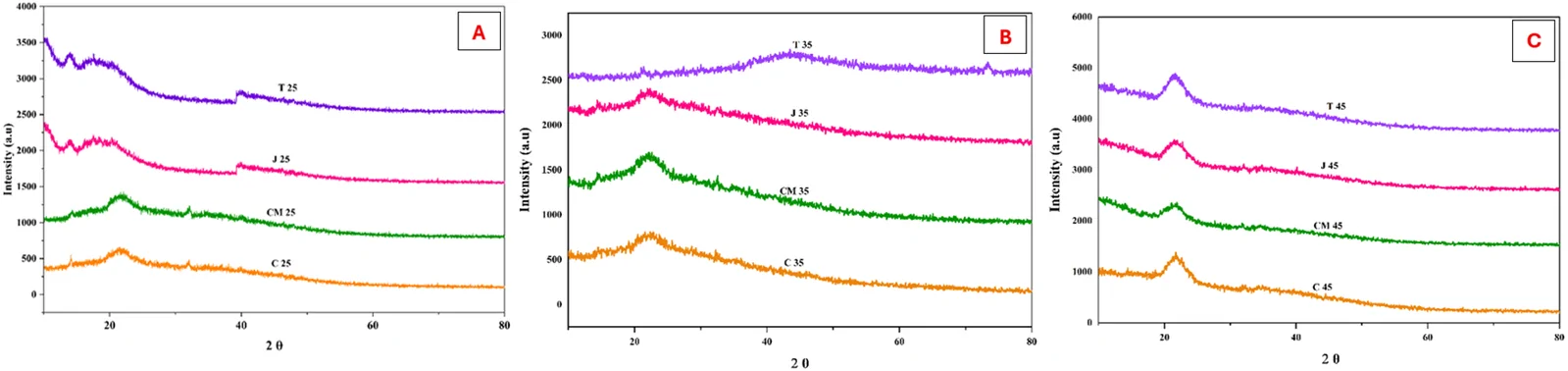
X-ray diffraction (XRD) patterns of dragon fruit peel from four *Selenicereus* spp. varieties harvested at different maturity stages (**A**-25^th^, **B**-35^th^, and **C**-45^th^ DAA).

The semi-crystalline structure of lignocellulosic materials was verified by the XRD patterns of the peel powders of the four varieties (J, CM, C, and T) at three maturity stages (25th, 35th, and 45th DAA) (**Figure 5**). All the samples had a wide diffraction peak with a center of 2*θ* = 20–22*θ*, which is associated with cellulose, exhibiting that the predominant crystalline component is cellulose (Qian et al., 2022). The varieties showed moderate peak intensity on the 25th DAA, with an amorphous background, suggesting a less-ordered arrangement of polysaccharides. Variety T showed higher intensity and contained more crystalline cellulose at an early maturity stage compared with the samples from varieties J, CM, and C. Micro-reflections at 2*θ*, 15*θ*, and 34*θ* also indicated the presence of cellulose microfibrils (Srinivasan and Rana, 2025). As maturity increased, the sharpness of the cellulose peak increased progressively. The higher intensities and less broadening of the peaks in the 45th DAA samples were greater than those at earlier stages, indicating better structural order and higher crystallinity. This tendency implies maturation-related reorganization of cell wall polysaccharides and depletion of the amorphous phase. Variety T was the most crystalline with the highest diffraction intensity, whereas variety C showed low absolute intensity, but sharp peaks at full maturity. The CM and J varieties exhibited intermediate behavior with crystalline domains maintained at various stages. The peak indicates the presence of amorphous components, such as hemicellulose and pectin, in the peel matrix. The gradual replacement of crystalline reflections by mature ones is a possible sign of a transition to a more organized cellulose structure. Overall, the XRD analysis showed that crystallinity increased with maturity across all varieties, in the following sequence: T > J > CM > C. These results verify that dragon fruit peel is mainly a crystalline cellulose network encased in an amorphous polysaccharide matrix, as reported for plant-based dietary fibers (He et al., 2020; Liu et al., 2019).

These structural characteristics indicate that dragon fruit peel consists of a semi-crystalline cellulose network embedded within an amorphous matrix of hemicellulose, pectin, and other polysaccharides. The increased dietary fiber content, microstructural porosity, and hydrophilic functional groups, observed concurrent with an increase in cellulose crystallinity with maturity, appeared to have a more profound impact on hydration behavior than crystallinity. Thus, despite the increase in cellulose crystallinity, mature peels exhibited improved WHC, SC, and OAC, suggesting that the increased porosity, dietary fiber content, and hydrophilic functional groups may outweigh the restrictive influence of crystallinity on hydration behavior. The structural organization and functional behavior of plant-based materials have been reported to be similar for variations in microstructure, polysaccharide arrangement, and molecular interactions in the hydration properties and ingredient functionality (Hou et al., 2024; Gong et al., 2024; Feng et al., 2025). These findings suggest that the enhanced functional performance of mature dragon fruit peel results from the combined effects of the compositional and structural modifications occurring during fruit maturation. In addition, with the advancement of analytical techniques, there is an improved understanding of the relationship between the compositional and structural characteristics and the antioxidant activity, physicochemical functionality, and potential food applications of plant materials (Feng et al., 2025; Quan et al., 2025; Chang et al., 2025).

In the present study, the use of hot air drying (55°C) resulted in a peel powder with the characteristics observed in the study. Thermal drying can lead to some degradation of the heat-sensitive bioactive compounds, such as phenolics and flavonoids, and can also affect the structure and properties of the cell walls, including their porosity and the organization of the polysaccharides (Lim et al., 2010; Coşkun et al., 2024; Rad et al., 2025). The use of alternative drying methods such as freeze drying, vacuum drying, and spray drying has been reported to minimize the degradation and oxidative damage during the drying process and to preserve bioactive compounds and antioxidant activity, along with structural integrity, in plant materials (Coşkun et al., 2024; Kasapoglu, 2025). Thus, the absolute values of antioxidant activity, functional properties, and microstructural characteristics of dragon fruit peel can be affected by the differences in the drying technology used. Additional comparative tests are needed to assess the effect of drying methods on the nutritional, structural, and functional quality of dragon fruit peel powder.

## 4 Conclusion

This study systematically evaluated the effects of maturity stage and genotype on the nutritional, functional, antioxidant, anti-nutritional, mineral, and structural characteristics of dragon fruit (*Selenicereus* spp.) peel. For several nutritional, functional, antioxidant, and mineral parameters, significant effects due to maturity stage, genotype, and their interactions were observed. Overall, mature peels (45th DAA) exhibited higher dietary fiber, minerals, WHC, OAC, and SC. However, immature peels (25th DAA) had higher levels of phenolics, flavonoids, and antioxidants. The SEM, FTIR, and XRD structural analyses showed that changes in the microstructure, functional groups, and cellulose organization are closely related to changes in the functional properties, which are associated with the maturity of the product. The results suggest that mature dragon fruit peel has favorable nutritional profile and technological properties. A decrease in antioxidant activity with maturity, however, indicated that the best harvest stage would depend on the intended use. Peels from the earlier maturity stages may be better for antioxidant applications. In contrast, mature peels might be preferred for use in food ingredient development, which may require improved hydration, swelling, and oil binding. In the present study, the nutrient availability and digestibility, toxicity, biological efficacy, consumer acceptance, and techno-economic feasibility of dragon fruit peel were not studied. More studies are needed to evaluate the effects of hot air oven drying on dragon fruit peel powder compared with other drying methods (e.g., freeze drying, spray drying, and vacuum drying). Thus, additional research on *in vitro* digestion, bioavailability studies, safety testing, formulation studies, and industrial-scale validation is needed to draw final conclusions on functional food, nutraceutical, and commercial uses. Further research is needed for *in vitro* digestion and bioavailability studies, biological activity validation, and the addition of dragon fruit peel powder in food formulations to assess its technological performance and consumer acceptability. Furthermore, techno-economic and sustainability analyses would enable the translation of dragon fruit peel valorization from laboratory-scale studies to industry.

## Data Availability

The raw data supporting the conclusions of this article will be made available by the authors, without undue reservation.
